# Supplementary material for: Personalized Treatment for Infantile Ascending Hereditary Spastic Paralysis Based on In Silico Strategies
Source: Molecules. 2022 Oct 19;27(20):7063. doi: 10.3390/molecules27207063 (PMC9609931; doi:10.3390/molecules27207063)
Supplement: Supplementary file 1 [file molecules-27-07063-s001.zip › molecules-1978299-supplementary.pdf]

Supporting information

Personalized Treatment for Infantile Ascending Hereditary Spastic Paralysis Based on In Silico Strategies

Matteo Rossi Sebastiano [1], Giuseppe Ermondi [1], Kai Sato [2], Asako Otomo [2], Shinji Hadano [2] and Giulia Caron [1,*]

[1] Molecular Biotechnology and Health Sciences Department, University of Torino, Quarello 15, 10135 Torino, Italy

[2] Molecular Neuropathobiology Laboratory, Department of Molecular Life Sciences, Tokai University School of Medicine, 143 Shimokasuya, Isehara, 259-1193, Japan

Table S1. PDB codes of the structures used for homology modeling.

Model	PDB templates
VPS9	1TXU, 2OT3
HB	2OT3
Linker	3LP8
Rab5a	2EFC

Table S2. ClusPro dimers energy and scoring.

Dimer	Cluster	Members	Representative	Weighted Score	Lowest Energy
2*WT-VPS9	0	67	Center	-1191.4	-1309
2*WT-VPS9	1	60	Center	-1023.7	-1258.2
2*WT-VPS9	2	59	Center	-1154.3	-1380.9
2*WT-VPS9	3	59	Center	-1001	-1272.1
2*WT-VPS9	4	49	Center	-1032	-1207.9
2*WT-VPS9	5	37	Center	-1047.2	-1298.7
2*WT-VPS9	6	37	Center	-1044.7	-1128.2
2*WT-VPS9	7	36	Center	-1042.8	-1135.4
2*WT-VPS9	8	34	Center	-1064.8	-1274
2*WT-VPS9	9	30	Center	-1203.1	-1203.1
2*WT-VPS9	10	26	Center	-1127.8	-1127.8
2*WT-VPS9	11	24	Center	-1061.2	-1258.2
2*WT-VPS9	12	23	Center	-1067.5	-1205.8
2*WT-VPS9	13	20	Center	-1023.3	-1143.8
2*WT-VPS9	14	19	Center	-1024.2	-1091.2
2*WT-VPS9	15	19	Center	-1083.7	-1230.4
2*WT-VPS9	16	16	Center	-1162.5	-1309.2
2*WT-VPS9	17	15	Center	-1059.1	-1059.1
2*WT-VPS9	18	14	Center	-1130.6	-1132.7
2*WT-VPS9	19	13	Center	-998	-1227.2
2*WT-VPS9	20	13	Center	-1063.1	-1097.7
2*WT-VPS9	21	12	Center	-1021	-1138.6
2*WT-VPS9	22	12	Center	-1009.7	-1143.6
2*WT-VPS9	23	12	Center	-1052.6	-1052.6
2*WT-VPS9	24	12	Center	-1038.9	-1086.8
2*WT-VPS9	25	12	Center	-1193.4	-1193.4
2*WT-VPS9	26	12	Center	-1096.8	-1114.1
2*WT-VPS9	27	11	Center	-1191.4	-1191.4
2*WT-VPS9	28	11	Center	-1181.5	-1181.5
2*WT-VPS9	29	11	Center	-1128.1	-1128.1

2*R1611W-VPS9	0	74	Center	-1113	-1341.8
2*R1611W-VPS9	1	55	Center	-946.1	-1228
2*R1611W-VPS9	2	39	Center	-1036.8	-1103.3
2*R1611W-VPS9	3	30	Center	-987.7	-1025.3
2*R1611W-VPS9	4	28	Center	-979.9	-1013.1
2*R1611W-VPS9	5	25	Center	-1008.6	-1008.6
2*R1611W-VPS9	6	24	Center	-1146.9	-1146.9
2*R1611W-VPS9	7	22	Center	-1018.7	-1063.8
2*R1611W-VPS9	8	22	Center	-976.4	-1059.3
2*R1611W-VPS9	9	19	Center	-926	-1002.5
2*R1611W-VPS9	10	17	Center	-1007.7	-1044.1
2*R1611W-VPS9	11	16	Center	-913.9	-994.8
2*R1611W-VPS9	12	15	Center	-943.2	-1067.6
2*R1611W-VPS9	13	15	Center	-1034.9	-1034.9
2*R1611W-VPS9	14	15	Center	-1032	-1050.1
2*R1611W-VPS9	15	15	Center	-981.9	-981.9
2*R1611W-VPS9	16	14	Center	-1061.6	-1061.6
2*R1611W-VPS9	17	14	Center	-980.6	-980.6
2*R1611W-VPS9	18	13	Center	-1003.3	-1003.3
2*R1611W-VPS9	19	13	Center	-962.6	-993.9
2*R1611W-VPS9	20	13	Center	-1008.3	-1008.3
2*R1611W-VPS9	21	13	Center	-915.8	-1078.2
2*R1611W-VPS9	22	12	Center	-916.6	-998.3
2*R1611W-VPS9	23	11	Center	-942.8	-1080.9
2*R1611W-VPS9	24	11	Center	-1034.2	-1034.2
2*R1611W-VPS9	25	11	Center	-1030.7	-1030.7
2*R1611W-VPS9	26	11	Center	-996	-996
2*R1611W-VPS9	27	11	Center	-971.9	-971.9
2*R1611W-VPS9	28	11	Center	-950.8	-1018.5
2*R1611W-VPS9	29	10	Center	-915.1	-1051.6
WT-VPS9/RLD	0	67	Center	-1121.4	-1322.2
WT-VPS9/RLD	1	60	Center	-899.7	-1011.5
WT-VPS9/RLD	2	52	Center	-980.7	-1120.2
WT-VPS9/RLD	3	51	Center	-923.6	-1087.5
WT-VPS9/RLD	4	46	Center	-925.4	-1035
WT-VPS9/RLD	5	36	Center	-1000.9	-1192.6
WT-VPS9/RLD	6	28	Center	-1005.9	-1005.9
WT-VPS9/RLD	7	28	Center	-884.5	-1026.4
WT-VPS9/RLD	8	27	Center	-870.3	-945.5
WT-VPS9/RLD	9	26	Center	-1092.7	-1092.7
WT-VPS9/RLD	10	24	Center	-1054.6	-1054.6
WT-VPS9/RLD	11	23	Center	-889.6	-1133
WT-VPS9/RLD	12	23	Center	-896.3	-1038.7
WT-VPS9/RLD	13	21	Center	-973.6	-1012.1
WT-VPS9/RLD	14	21	Center	-973	-973
WT-VPS9/RLD	15	20	Center	-995.8	-1174.3
WT-VPS9/RLD	16	20	Center	-944.5	-1041.9
WT-VPS9/RLD	17	20	Center	-961.7	-1020.2
WT-VPS9/RLD	18	18	Center	-902.8	-987.6
WT-VPS9/RLD	19	18	Center	-906.2	-1056.7
WT-VPS9/RLD	20	17	Center	-882.7	-1041
WT-VPS9/RLD	21	17	Center	-967.1	-967.1
WT-VPS9/RLD	22	16	Center	-995.7	-995.7
WT-VPS9/RLD	23	16	Center	-959.4	-959.4

WT-VPS9/RLD	24	15	Center	-1017.3	-1017.3
WT-VPS9/RLD	25	13	Center	-1008.3	-1092.2
WT-VPS9/RLD	26	13	Center	-962	-962
WT-VPS9/RLD	27	13	Center	-915.9	-954.6
WT-VPS9/RLD	28	11	Center	-942.7	-950.2
WT-VPS9/RLD	29	10	Center	-901.5	-917.1
R1611W-VPS9/RLD	0	51	Center	-924.4	-1006.4
R1611W-VPS9/RLD	1	49	Center	-950.5	-1162.9
R1611W-VPS9/RLD	2	37	Center	-996.1	-1065.9
R1611W-VPS9/RLD	3	32	Center	-942.2	-1016.5
R1611W-VPS9/RLD	4	30	Center	-862	-975.6
R1611W-VPS9/RLD	5	29	Center	-957.8	-985.9
R1611W-VPS9/RLD	6	27	Center	-965.9	-1112.5
R1611W-VPS9/RLD	7	27	Center	-898.4	-1029.6
R1611W-VPS9/RLD	8	27	Center	-961	-961
R1611W-VPS9/RLD	9	26	Center	-968.8	-968.8
R1611W-VPS9/RLD	10	25	Center	-927.5	-1067.3
R1611W-VPS9/RLD	11	24	Center	-904	-1053.3
R1611W-VPS9/RLD	12	23	Center	-967.6	-967.6
R1611W-VPS9/RLD	13	22	Center	-869.5	-973.9
R1611W-VPS9/RLD	14	22	Center	-875.2	-976.8
R1611W-VPS9/RLD	15	22	Center	-948.5	-948.5
R1611W-VPS9/RLD	16	20	Center	-864.6	-951
R1611W-VPS9/RLD	17	20	Center	-882.5	-1000.1
R1611W-VPS9/RLD	18	20	Center	-877.1	-913.5
R1611W-VPS9/RLD	19	19	Center	-997.4	-997.4
R1611W-VPS9/RLD	20	18	Center	-961	-961
R1611W-VPS9/RLD	21	18	Center	-924.9	-924.9
R1611W-VPS9/RLD	22	17	Center	-941.7	-1024.9
R1611W-VPS9/RLD	23	15	Center	-876.1	-1010.7
R1611W-VPS9/RLD	24	15	Center	-948.7	-976.8
R1611W-VPS9/RLD	25	14	Center	-858.2	-919.4
R1611W-VPS9/RLD	26	14	Center	-903.2	-964.7
R1611W-VPS9/RLD	27	13	Center	-938.6	-987.7
R1611W-VPS9/RLD	28	13	Center	-882.7	-902.2
R1611W-VPS9/RLD	29	11	Center	-844.5	-913.5

Table S3. Interaction energy of the dimer models calculated with AMBER 12:EHT forcefield.

Model number	2*WT-VPS9	2*R1611W-VPS9	WT-VPS9/RLD	R1611W-VPS9/RLD
0	-70.889	20.2	-350.945	-380.701
1	-320.304	-296.215	-276.586	-188.63
2	-341.068	-276.39	-201.834	-332.963
3	-199.396	-230.703	-453.573	-444.925
4	-54.765	-439.247	82.518	-260.352
5	-233.646	342.643	-361.772	-478.659
6	-206.456	-102.496	-463.178	-328.447
7	-83.696	-299.288	415.538	-336.713
8	-330.278	-295.056	-163.902	-145.319
9	-278.787	-229.267	-380.889	-502.481
10	-328.3	-162.77	-163.156	-194.201
11	-197.615	-348.842	-392.858	-321.348
12	-178.949	-94.909	-209.578	-167.95
13	-276.083	-257.679	-310.578	-318.944

14	-203.865	-78.856	-73.356	-295.192
15	-123.623	-187.537	-298.773	-396.191
16	-277.326	-170.581	-215.8	-329.553
17	-202.451	-228.791	-449.538	-325.37
18	137.317	-225.864	-377.78	84.454
19	-348.813	-213.038	-265.599	-188.156
20	-185.83	-419.488	-385.232	-276.66
21	-115.683	-278.6	-461.568	-174.541
22	336.884	-377.219	-332.81	-318.801
23	-267.707	-125.276	-110.954	-271.703
24	-396.473	-349.511	-195.59	-608.401
25	-209.497	-343.875	-373.636	-280.112
26	-174.397	-157.101	-144.509	-246.091
27	-328.068	-303.536	-246.454	-348.078
28	-237.256	-259.607	-280.215	-629.951
29	-185.714	-180.094	-517.442	-305.129

Table S4. ΔΔG computed values.

Model number	2*WT-VPS9	2*R1611W-VPS9	WT-VPS9/RLD	R1611W-VPS9/RLD
0	0.953794	1.713622	0.135349	-2.686
1	0.76082	-3.8141	0.029245	-2.9556
2	0.872542	1.939402	0.029838	-0.90358
3	1.129841	1.38663	0.14839	-2.681
4	0.916554	2.095112	0.153725	-2.21168
5	1.630898	-0.63761	0.036951	-0.92854
6	0.886084	-0.83224	0.029838	-2.12181
7	0.164968	1.760335	0.029245	-2.76089
8	0.791289	-2.14021	0.029245	-0.95351
9	0.117571	-1.4473	0.072517	-0.97348
10	0.466279	-1.67308	0.029245	-0.93354
11	0.354557	1.822619	0.029245	-0.90857
12	0.212365	1.83819	0.029838	-0.96849
13	1.011348	-0.82446	0.029245	-1.76733
14	0.385027	-0.76996	0.029245	-3.07543
15	0.14127	1.713622	0.029245	-3.3051
16	0.151426	1.962758	0.14187	-2.09186
17	1.160311	1.246491	0.192254	-0.97348
18	0.04986	-1.5018	0.029245	-1.00344
19	0.909783	-1.54073	0.077851	-0.96349
20	0.164968	1.557911	0.139498	-1.24309
21	0.232679	0.48351	0.074888	-0.99345
22	0.137884	-0.82446	0.030431	-0.9585
23	0.107414	1.807048	0.029838	-3.38997
24	0.313931	1.853761	0.029245	-0.96849
25	1.062131	0.094234	0.093856	-3.35503
26	0.198823	-0.81667	0.029245	-3.3001
27	-0.02124	1.845976	0.196996	-0.93853
28	0.131113	2.009472	0.029245	-0.94851
29	1.441309	1.067424	0.029838	-1.44779

Table S5. Contact residues of the VPS9/Rab5 interaction complex.

Rab5a residue	Number	Alsin residue	Number
Ser	34	Trp	1596

Leu	38	Trp	1596
Glu	50	Glu	1636
Thr	52	Met	1640
Thr	52	Thr	1643
Ile	53	Ile	1575
Ile	53	Thr	1578
Ile	53	Met	1640
Ile	53	Thr	1643
Ala	55	Ser	1583
Ala	56	Leu	1601
Ala	56	Phe	1605
Ala	56	Thr	1643
Asp	75	Met	1598
Thr	76	Met	1598
Thr	76	Asp	1599
Ala	77	Asp	1599
Ala	77	Met	1598
Ala	77	Asp	1600
Ala	77	Ser	1597
Gln	79	Asp	1599
Ala	86	Ala	1646
Ala	86	Cys	1647
Met	88	Thr	1642
Met	88	Ala	1646

Table S6. Effects of MK4 treatment on the distribution of Alsin in HeLa cells. WT: Alsin^{WT}, R1611W: Alsin^{R1611W}, TrioD: Trio-GEFD1, MK4: Menaquinone 4.

	MK4	Number of counted cells	Endosomal localization		Aggregate formation	
		(n)	(n)	(%)	(n)	(%)
WT	-	33	6	18.2	0	0.0
	+	39	11	28.2	0	0.0
WT / TrioD	-	42	28	66.7	0	0.0
	+	58	55	94.8	1	1.7
R1611W	-	13	0	0.0	0	0.0
	+	33	0	0.0	1	3.0
R1611W / TrioD	-	25	0	0.0	4	16.0
	+	23	4	17.4	2	8.7

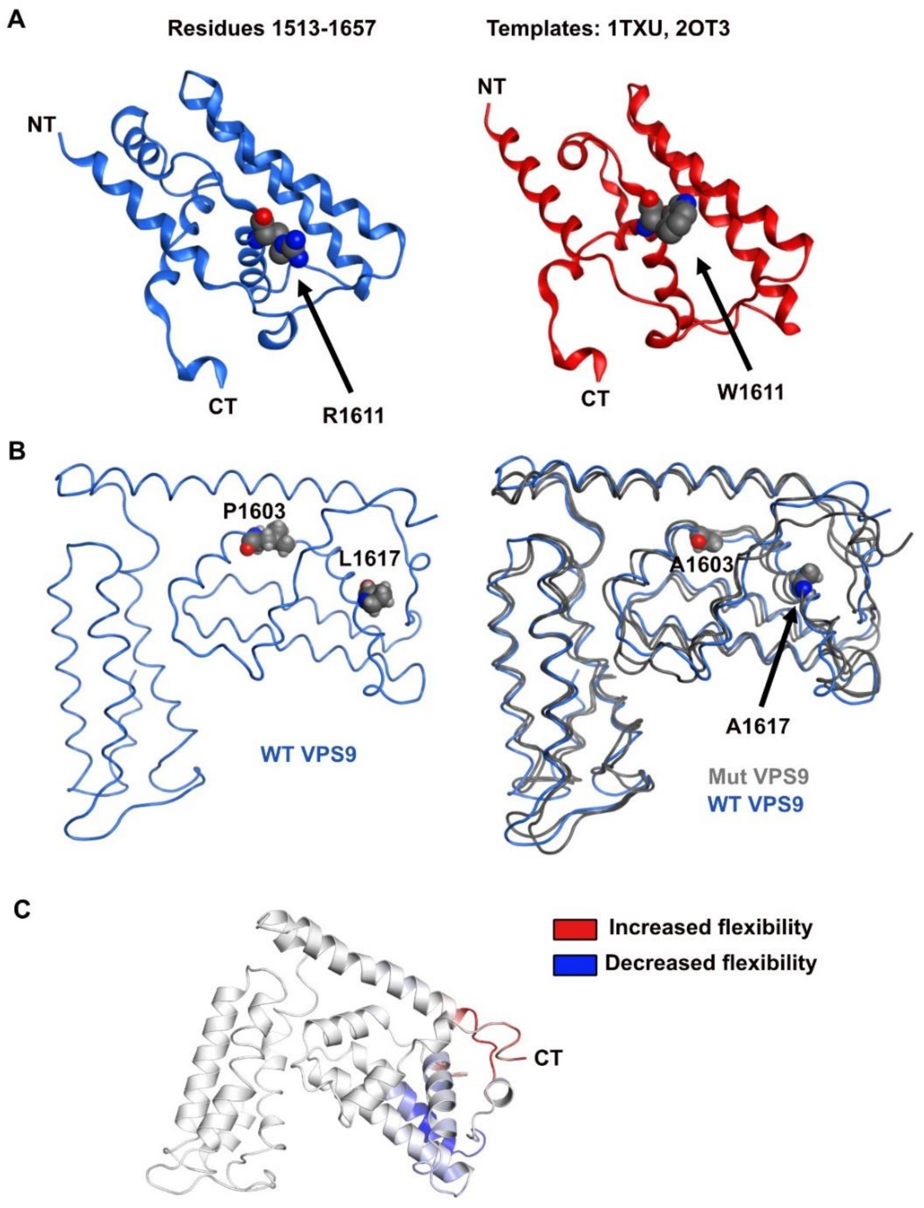

Figure S1. Modeling and stability testing of VPS9. A) WT (left) and R1611W (right) models of VPS9-core B) WT (left, blue) VPS9-HB, residues P1603 and L1617 are highlighted. Mutated (right, grey) VPS9-HB superposed to the WT (blue) structure; mutated residues (either P1603A or 1617A) are highlighted. C) Normal mode analysis for mutation R1611W on a WT VPS9-HB model.

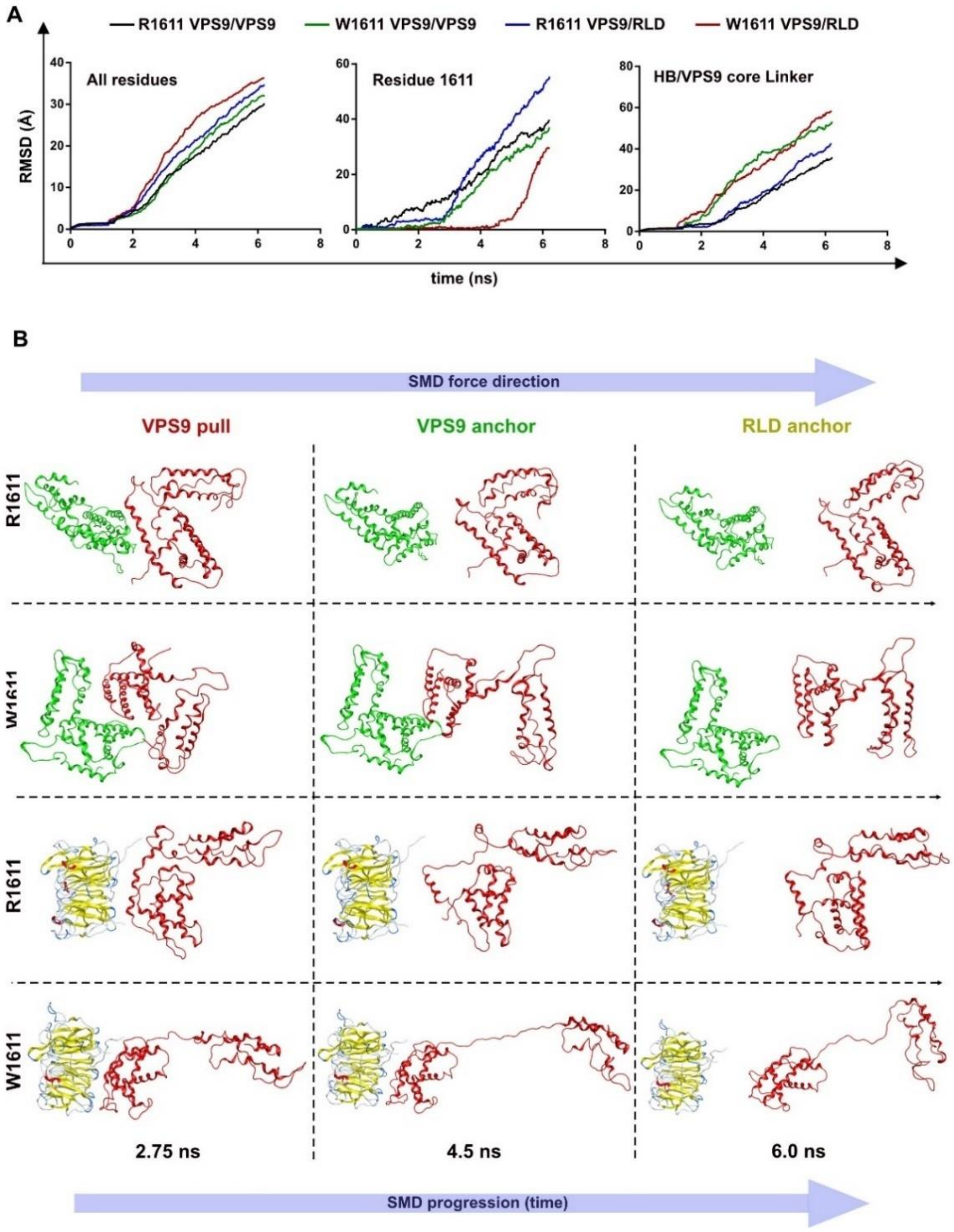

Figure S2. Assessment of dimers stability via SMD. A) RMSD plots of different residue selections from SMD in Figure 5C B) Snapshots at different stages from SMD in Figure 5C.

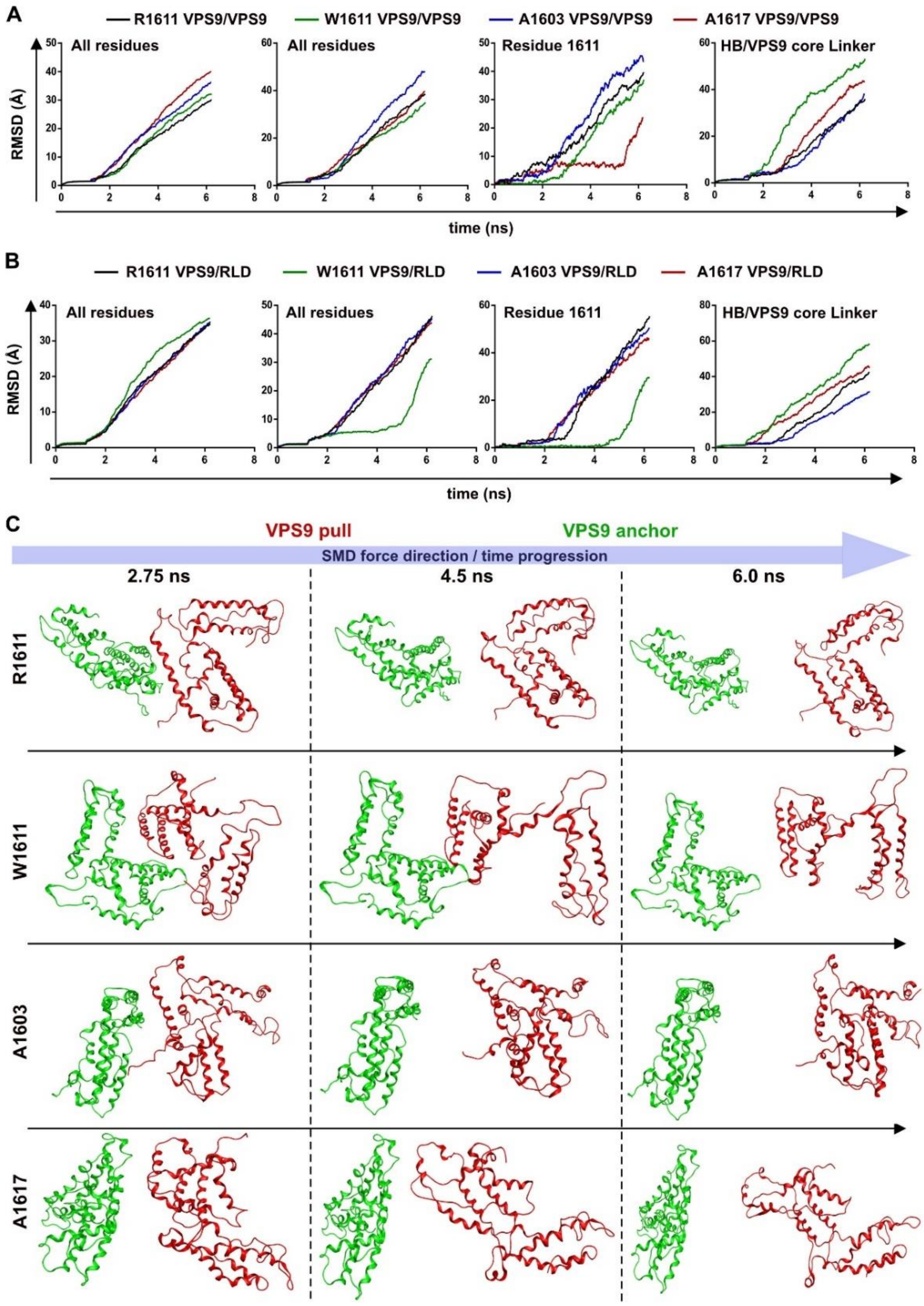

Figure S3. Strategy verification. A, B) RMSD time-course plots of different residue selections from SMD of VPS9 homodimers (A) and VPS9/RLD when mutations 1603A and 1617A are introduced. C) Relevant snapshots of VPS9 homodimers at different temporal stages from SMD in A, B.

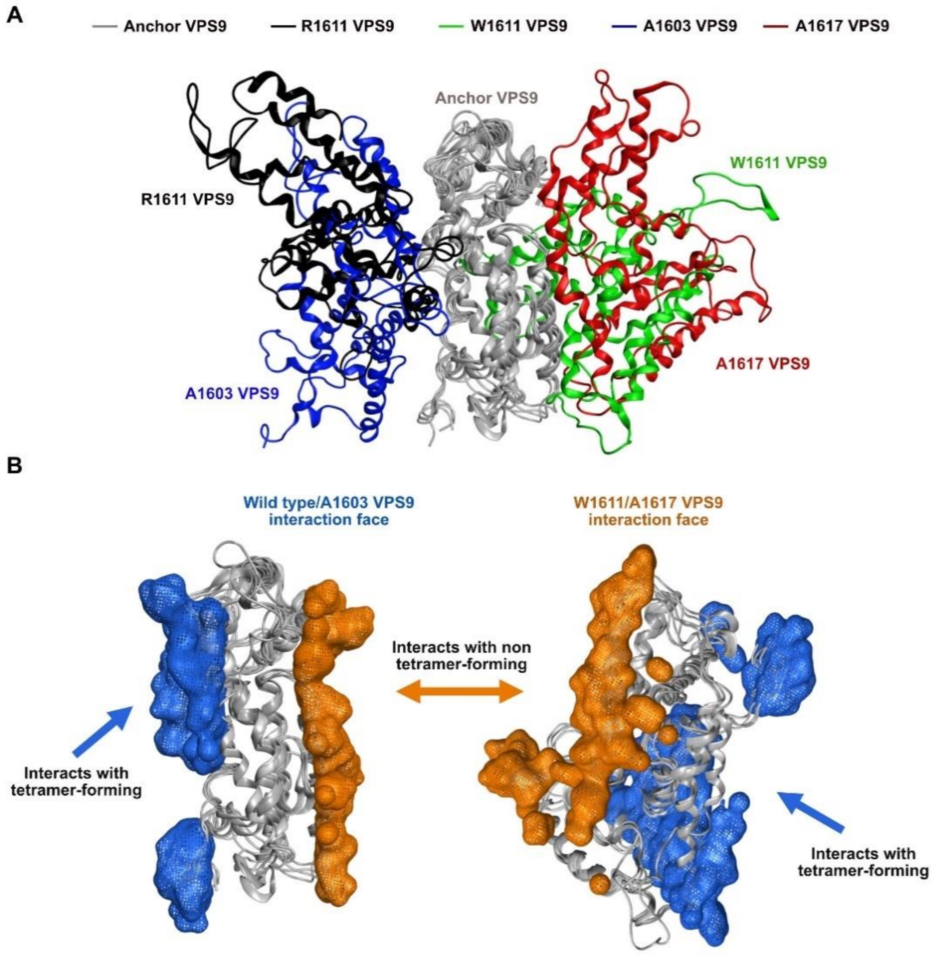

Figure S4. Strategy verification II. A) Superposition of initial VPS9 homodimers, VPS9/RLD heterodimers with mutations at site 1611, 1603 and 1617 utilized as starting point for SMD simulations in Figure 5, S2 and S3. B) Interaction surface (for representation purpose) of VPS9 homodimers for tetramer-forming species (WT and 1603A) and non-tetramer-forming species (1611W and 1617A).

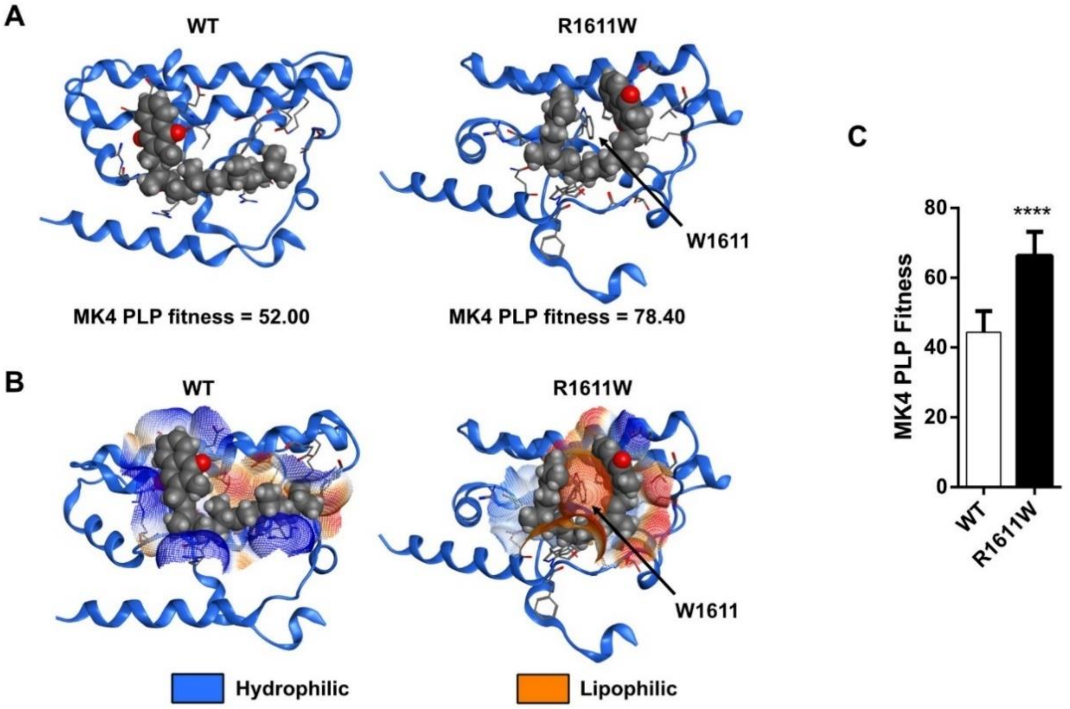

Figure S5. MK4 docks to mutated VPS9 with higher affinity. A) Best scored docking pose of MK4 to wt and R1611W VPS9.B) Interaction surface of VPS9/MK4 interaction site highlighting the higher hydrophobic nature of MK4 interaction to mutated VPS9.C) Comparison of PLP fitness docking score (the higher the score, the higher the affinity of the ligand) from the first 10 docking poses from panel S5A.

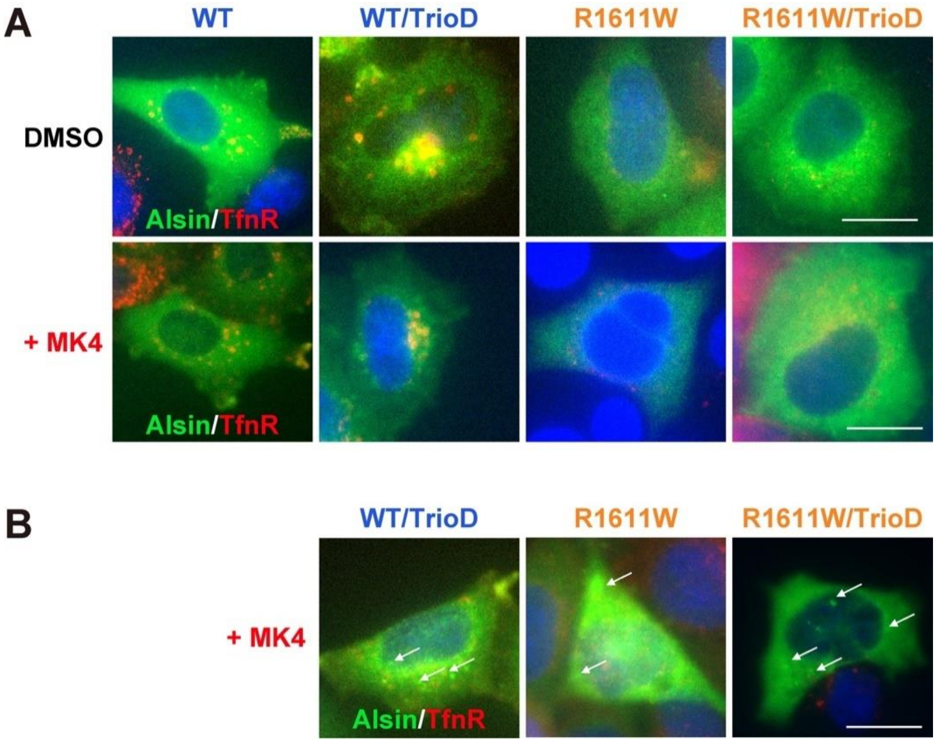

Figure S6. Effects of MK4 treatment on the distribution of Alsin in HeLa cells. A and B) HeLa cells were transfected with pCIneoFLAG–ALS2^{WT} (WT) or pCIneoFLAG–ALS2^{R1611W} (R1611W). To activate endogenous Rac1 thereby facilitating endosomal localization of Alsin, the cells were co-transfected Alsin construct with pCIneoHA-Trio-GEFD1 (1233–1628) (TrioD). Six hours of transfection, the cells were treated with 10 µM MK4 for 24 hr. After fixation, the cells were stained with anti-FLAG (green; Alsin) and anti-transferrin receptor (Red; TnfR) antibodies. Representative merged images are shown: Alsin colocalized with TfnR (yellow signals) represents endosome-localizing Alsin. B) Arrows indicate Alsin-positive aggregates. Scale bars, 20 µm.
